# Evolution and prognostic implications of cardiac damage in women after transcatheter aortic valve implantation

**DOI:** 10.1007/s10554-025-03424-8

**Published:** 2025-05-22

**Authors:** Rinchyenkhand Myagmardorj, Federico Fortuni, Xavier Galloo, Takeru Nabeta, Maria Chiara Meucci, Steele C. Butcher, Frank van der Kley, Nina Ajmone Marsan, Jeroen J. Bax

**Affiliations:** 1 Department of Cardiology, Heart Lung Centre, Leiden University Medical Centre (LUMC), Albinusdreef 2, 2300 RC Leiden, The Netherlands; 2 Cardiology and Cardiovascular Pathophysiology, S. Maria Della Misericordia Hospital, University of Perugia, Perugia, Italy; 3 Heart Center, University of Turku and Turku University Hospital, Turku, Finland

**Keywords:** Aortic stenosis, Valvular heart disease, Prognosis, Risk assessment, Transcatheter aortic valve implantation, Women

## Abstract

Recently, a cardiac damage staging system has been proposed in patients with severe AS to improve risk stratification, but there is still paucity of data in women. Accordingly, we aimed to characterize the change in cardiac damage after transcatheter aortic valve implantation (TAVI), and to assess the prognostic value of cardiac damage staging in women. A total of 334 women (mean age 81 ± 7 years) with severe AS undergoing TAVI were included and retrospectively analyzed. Echocardiography was performed before and 6 months after TAVI. Patients were classified according to the following stages of cardiac damage: 0 = no damage; 1 = left ventricular damage; 2 = left atrial or mitral valve damage; 3 = pulmonary vasculature or tricuspid valve damage; and 4 = right ventricular damage. The primary endpoint was all-cause mortality. Most patients presented with advanced heart failure symptoms (62% in NYHA III-IV). TAVI consistently improved cardiac damage of at least one stage in 43% of patients at 6-month follow-up. During a median follow-up of 48 months, 79 patients (24%) died. Each increment of both baseline (HR per 1-stage increment 1.537, P = 0.001) and follow-up (HR per 1-stage increment 1.714, P = 0.01) cardiac damage stage were independently associated with all-cause death. Moreover, the re-assessment of cardiac damage at 6-month follow-up provided incremental prognostic value over baseline assessment (Chi-square change = 6.885; P = 0.009). TAVI has a beneficial effect on cardiac function and remodeling in women. Cardiac damage assessed before and 6 months after TAVI showed to be consistently and independently associated with prognosis.

**Supplementary Information:**

The online version contains supplementary material available at 10.1007/s10554-025-03424-8.

## Introduction

Aortic stenosis (AS) is the most common valvular heart disease in developed countries, with a global prevalence of 9.4 million in 2019 (1). Aortic valve replacement (AVR) is the only available treatment for severe AS and includes surgical and transcatheter aortic valve implantation (TAVI). TAVI showed to be safe and effective for the treatment of severe AS across all categories of surgical risk, from low- to high-risk patients (2–4). Although current guidelines recommend either surgical or transcatheter AVR for patients with severe AS with AS-related symptoms and/or left ventricular (LV) systolic dysfunction (5), an extra-valvular cardiac damage staging including the assessment of the LV, left atrium (LA), mitral regurgitation (MR), pulmonary pressures, tricuspid regurgitation (TR), and right ventricle (RV) had been proposed in severe AS to optimize risk stratification and identify the best timing for AVR (6, 7).

Recent studies (8–12) on AS suggested morphological and clinical outcome differences between men and women. Since women and men have different anatomical and pathophysiologic features relative to AS and AS-related cardiac remodeling, they exhibit different AS-related phenotypes (8–10). Although women have more peri-procedural complications they show better survival rates after TAVI, probably due to a lower burden of cardiovascular risk factors compared to men (11, 12). As compared with men, women have smaller aortic annulus size and different morphology of AS, requiring tailored therapy mostly based on imaging assessment (8–10). Considering these sex-specific differences, recent trials (e.g., the VIVA [Transcatheter Aortic Valve Replacement Versus Surgical Aortic Valve Replacement for Treating Elderly Patients with Severe Aortic Stenosis and small Aortic Annuli; NCT03383445] and RHEIA trial [Randomized researcH in womEn all comers wIth Aortic stenosis; NCT04160130]) (13, 14) were specifically designed to assess the efficacy and safety of transcatheter versus surgical AVR in women. Since there is still paucity of data about extra-valvular cardiac damage classification and its evolution after TAVI in women, the aim of the current study was to identify the eventual change in the extent of cardiac damage after TAVI, and to evaluate the prognostic value of cardiac damage staging at baseline and follow-up in women with severe AS undergoing TAVI.

## Methods

### Patient population and clinical data

Female patients with severe AS aged above 18 years old undergoing TAVI at the Leiden University Medical Center (Leiden, The Netherlands) between November 2007 and December 2019 were included and retrospectively analyzed (15). AS severity was defined according to contemporary guidelines, and severe AS was diagnosed when the aortic valve area (derived using the continuity equation) was < 1.0 cm^2^ (or an indexed aortic valve area < 0.6 cm^2^/m^2^), mean aortic valve gradient ≥ 40 mmHg, and/or peak aortic jet velocity ≥ 4 m/s (16). Eligibility and feasibility of TAVI were decided by the local heart team. Patients with congenital heart disease, heart transplantation, supra- or sub-valvular AS, dynamic LV outflow tract obstruction, infectious endocarditis or previous valve-in-valve procedure were excluded (Supplementary Fig. 1S). Four authors (R.M., F.F., N.A.M., and J.J.B.) were involved in the patient selection process, and disagreement was resolved with discussion and consensus among them. Patients were evaluated using transthoracic echocardiography (TTE) to assess the severity of AS and extra-valvular cardiac damage.

Demographic, clinical, and laboratory data were collected from the departmental patient information system (EPD-Vision; Leiden University Medical Center, Leiden, The Netherlands) and hospital records (HiX; ChipSoft, Amsterdam, The Netherlands) at the closest time point before the baseline pre-TAVI echocardiographic assessment. Clinical characteristics included symptoms, cardiovascular risk factors, comorbidities and medication. Body surface area (BSA), body mass index, and European System for Cardiac Operative Risk Evaluation (EuroSCORE) were assessed as recommended. This retrospective analysis of clinically acquired data complied with the STROBE guidelines (Supplementary Table 1S) and was approved by the institutional review board of the Leiden University Medical Center (DAP/tak/1182024), and due to the retrospective nature of the data, the need for patient written informed consent was waived.

### Transthoracic echocardiography

All TTE examinations were performed at baseline and 6 months after TAVI. Echocardiographic data were obtained using available ultrasound systems (Vivid 7 and E9 systems; General Electric Vingmed, Horten, Norway) equipped with 3.5 MHz or M5S transducers. All images were digitally stored for offline analysis using commercially available software (EchoPAC versions 203 and 204; GE Medical Systems, Vingmed, Horten, Norway). From parasternal, apical, and subcostal views, M-mode, two-dimensional, color-, continuous wave-, and pulsed wave Doppler data were acquired in accordance with current guidelines (17–20). From the parasternal long-axis view, LV dimensions were measured, and LV mass was calculated based on Devereux’s formula and indexed to BSA (17). Also, relative wall thickness (RWT) was calculated using the recommended formula [(2*posterior wall thickness)/LV internal diameter at end-diastole] (17). LV volumes were measured from the apical 4- and 2-chamber views using the biplane Simpson’s method, and LV ejection fraction was calculated. Left atrial volumes were measured using the biplane method of disks from the apical 4- and 2-chamber views and indexed to BSA (17). Mitral and tricuspid regurgitation severity were assessed using a multiparametric approach as suggested by current guidelines, taking into account qualitative, semi-quantitative and quantitative parameters (18). From the apical 4-chamber view, peak early (E) and late (A) diastolic velocities were measured using pulsed-wave Doppler recordings of the transmitral flow (19). The e’ was measured at both lateral and septal sides of the mitral annulus using tissue Doppler imaging and averaged to calculate the E/e’ ratio. Pulmonary artery systolic pressures (PASP) were estimated from the maximum TR velocity by applying the Bernoulli equation and adding 3, 8, or 15 mmHg based on the diameter and collapse of the inferior vena cava (20). Tricuspid annular planar systolic excursion (TAPSE) was measured using M-mode recordings of the lateral tricuspid annulus acquired from an RV-focused apical 4-chamber view (20). All ventricular and atrial measurements were indexed to BSA. Aortic peak and mean transvalvular gradients were derived from the apical 3- or 5-chamber views using the Bernoulli equation. The aortic valve area was calculated using the continuity equation and indexed to BSA (16).

Patients were classified into 5 distinct stages based on the presence of cardiac damage (6, 7). This staging system included stage 0 = no signs of cardiac damage; stage 1 = LV damage identified as LVEF < 50% and/or *E*/e´ > 14 and/or LV mass index (LVMI) > 95 g/m^2^; stage 2 = mitral valve or LA damage identified as moderate or severe MR and/or indexed LA volume > 34 mL/m^2^; stage 3 = pulmonary vasculature or tricuspid valve damage identified as PASP ≥ 60 mmHg and/or moderate or severe TR; and stage 4 = RV damage identified as TAPSE < 17 mm (Supplementary Fig. 2S). Notably, considering the very low likelihood of permanent atrial fibrillation (AF) reversibility after TAVI, AF was not included in the definition of cardiac damage stage 2 (7). The cardiac damage staging system was applied hierarchically meaning that a patient at a specific stage may encompass all or only some criteria of that stage as well as criteria from preceding stages. For instance, a patient with RV dysfunction (Stage 4) could also present with LV dysfunction, secondary MR, or pulmonary hypertension. Similarly, patients classified as Stage 3 (pulmonary hypertension or TV damage) often exhibit overlapping features but not specific characteristics that are present in the definition of more advanced stages, such as RV dysfunction (Stage 4). This hierarchical approach allows the cardiac damage staging system to provide a rational and holistic assessment of cardiac damage in severe AS, grounded in pathophysiology.

### Follow-up and study endpoint

Patients were followed up for the occurrence of the primary endpoint of all-cause mortality after TAVI. The follow-up duration was censored at 4 years. The survival data were collected from the departmental cardiology information system, which is linked to the municipal civil registries.

### Statistical analysis

Continuous variables were presented as mean ± SD if normally distributed and as median and interquartile range ([IQR], 25–75%) if non-normally distributed. To compare the variables at baseline and follow-up, the paired sample *T*-test and Wilcoxon signed-rank test were performed for continuous variables, and the McNemar test was used for categorical variables. A total of 29 patients (9%) died within 6 months after the procedure and therefore were not considered in the paired analysis (Supplementary Fig. 1S). Kaplan–Meier curves were created to estimate the 4-year survival rates. The log-rank test was used to analyze the differences in survival rates across the cardiac damage stages. Patients in stages 0 and 1 were merged into one stage at baseline and follow-up due to the small number of patients presenting with these stages of cardiac damage. Uni- and multivariable Cox regression analyses were performed to assess baseline clinical and echocardiographic variables independently associated with all-cause mortality. Statistically significant variables (P < 0.05) at the univariable Cox regression analysis were selected to be included in the multivariable Cox regression models. To assess associations between all-cause death and cardiac damage staging at follow-up, a landmark analysis was performed (21). Correlation coefficients were calculated to examine the presence of collinearity between the variables included in the multivariable Cox regression models and values below 0.5 were considered weak underlining lack of significant collinearity. Hazard ratios (HR) and 95% confidence intervals (CI) were calculated and reported. A two-sided P-value < 0.05 was considered significant. All data were analyzed with SPSS for Windows, version 29 (IBM SPSS Inc., IBM Corporation, Armonk, NY, USA).

## Results

A total of 334 women with severe AS undergoing TAVI were included (Supplementary Fig. 1S). Supplementary Table 2S shows the clinical characteristics of the total study population. The mean age was 81 ± 7 years; the majority of patients had hypertension (76%), dyslipidemia (60%), presented with New York Heart Association (NYHA) class III-IV heart failure symptoms (62%), and used cardiovascular medications including angiotensin-converting enzyme inhibitors or angiotensin II receptor blockers (56%), beta blockers (59%), diuretics (58%), and statins (56%).

Table 1 shows the echocardiographic characteristics of the cohort at baseline and follow-up. Six months after TAVI, LV mass and volumes decreased, whereas all individual indices of LV and RV systolic function improved, and pulmonary pressures decreased. In addition, the proportions of concomitant significant mitral (21% versus 15%) or tricuspid regurgitation (48% versus 29%) significantly decreased at follow-up. In terms of LV remodeling, a significant number of patients reversed concentric remodeling (24% versus 17%) and concentric hypertrophy (62% versus 40%), and the percentage of patients with normal LV geometry increased (from 4 to 13%).

**Table 1:** Baseline and follow-up echocardiographic characteristics after TAVI

Echocardiographic variables	Patients (n = 305)		P-value
	Baseline	6 months follow-up	
LV parameters			
LV end-diastolic diameter indexed (mm/m^2^)	25.1 ± 4.4	26.3 ± 3.9	** < 0.001**
LV end-systolic diameter indexed (mm/m^2^)	17.4 ± 5.1	17.4 ± 3.9	0.959
Relative wall thickness (mm)	0.6 ± 0.2	0.5 ± 0.1	**0.020**
LV end-diastolic volume (ml/m^2^)	45.7 ± 16.2	42.8 ± 12.9	**0.002**
LV end-systolic volume (ml/m^2^)	20.3 ± 12.8	16.2 ± 9.5	** < 0.001**
LV mass index (g/m^2^)	118.0 ± 37.4	110.8 ± 28.3	**0.001**
LV ejection fraction (%)	57.9 ± 12.6	63.1 ± 11.9	** < 0.001**
LV ejection fraction < 50%, n (%)	62 (21)	37 (13)	** < 0.001**
LV global longitudinal strain (%)	14.3 ± 4.1	17.4 ± 4.4	** < 0.001**
Moderate or severe mitral regurgitation, n (%)	62 (21)	44 (15)	**0.025**
E/e’ ratio	21.1 ± 13.2	25.6 ± 19.6	** < 0.001**
E/e′ > 14, n (%)	202 (70)	235 (81)	** < 0.001**
Left atrial parameters			
Left atrial volume index (ml/m^2^)	44.5 ± 16.2	43.9 ± 17.7	0.507
Left atrial volume index > 34 ml/m^2^, n (%)	215 (73)	196 (67)	**0.037**
RV parameters			
Systolic pulmonary arterial pressure (mmHg)	35.8 ± 14.9	31.8 ± 13.0	** < 0.001**
Systolic pulmonary arterial pressure ≥ 60 mmHg, n (%)	19 (6)	9 (3)	**0.041**
Moderate or severe tricuspid regurgitation, n (%)	145 (48)	88 (29)	** < 0.001**
Tricuspid annular plane systolic excursion (mm)	19.3 ± 4.2	20.1 ± 2.8	** < 0.001**
TAPSE < 17 mm, n (%)	56 (18)	15 (5)	** < 0.001**
Aortic valve parameters			
Mean aortic valve gradient (mmHg)	43.6 ± 18.8	10.3 ± 4.7	** < 0.001**
Peak aortic jet velocity (m/s)	4.0 ± 0.9	2.1 ± 0.5	** < 0.001**
LV remodeling patterns			
Normal geometry, n (%)	13 (4)	40 (13)	** < 0.001**
Concentric remodeling, n (%)	71 (24)	52 (17)	
Eccentric hypertrophy, n (%)	30 (10)	88 (30)	
Concentric hypertrophy, n (%)	184 (62)	119 (40)	

Continuous variables are presented as mean ± SD. Categorical variables are expressed as number (percentage)

*LV* Left ventricular, *TAPSE* Tricuspid annular planar systolic excursion

*P-values represent differences between stages of cardiac damage and are calculated by paired sample *T*-test test for continuous data and by McNemar test for categorical data

Bold values represent significant P-values

**Fig. 1:**
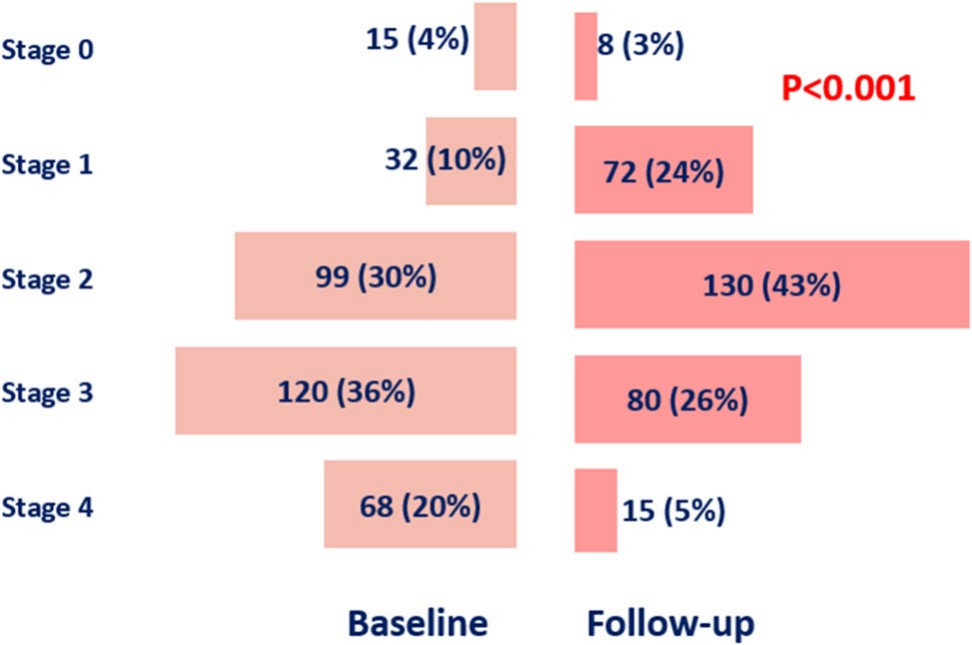
Cardiac damage distribution at baseline and follow-up. The frequency of patients (n, %) allocated for cardiac damage stages is shown at baseline and 6-month follow-up. Compared to baseline, there was a significant change (P < 0.001) in the distribution of staging at 6-month follow-up, with a decrease of patients in stages 3 and 4 as well as a concomitant increase in stages 1 and 2, demonstrating the favorable effect of TAVI on cardiac remodeling

Figure 1 shows the distribution of cardiac damage before and 6 months after TAVI. The proportions of cardiac damage stages were significantly different at follow-up as compared to baseline (P < 0.001), with a decrease of patients in stage 3 (pulmonary vasculature or tricuspid valve damage) and stage 4 (RV damage) and a concomitant increase of patients in stages 1 (LV damage) and 2 (LA or mitral valve damage). When considering the evolution of cardiac damage after TAVI, this regressed at least one stage in 43% of patients confirming the beneficial effect of TAVI on cardiac remodeling (Supplementary Fig. 3S).

**Fig. 2:**
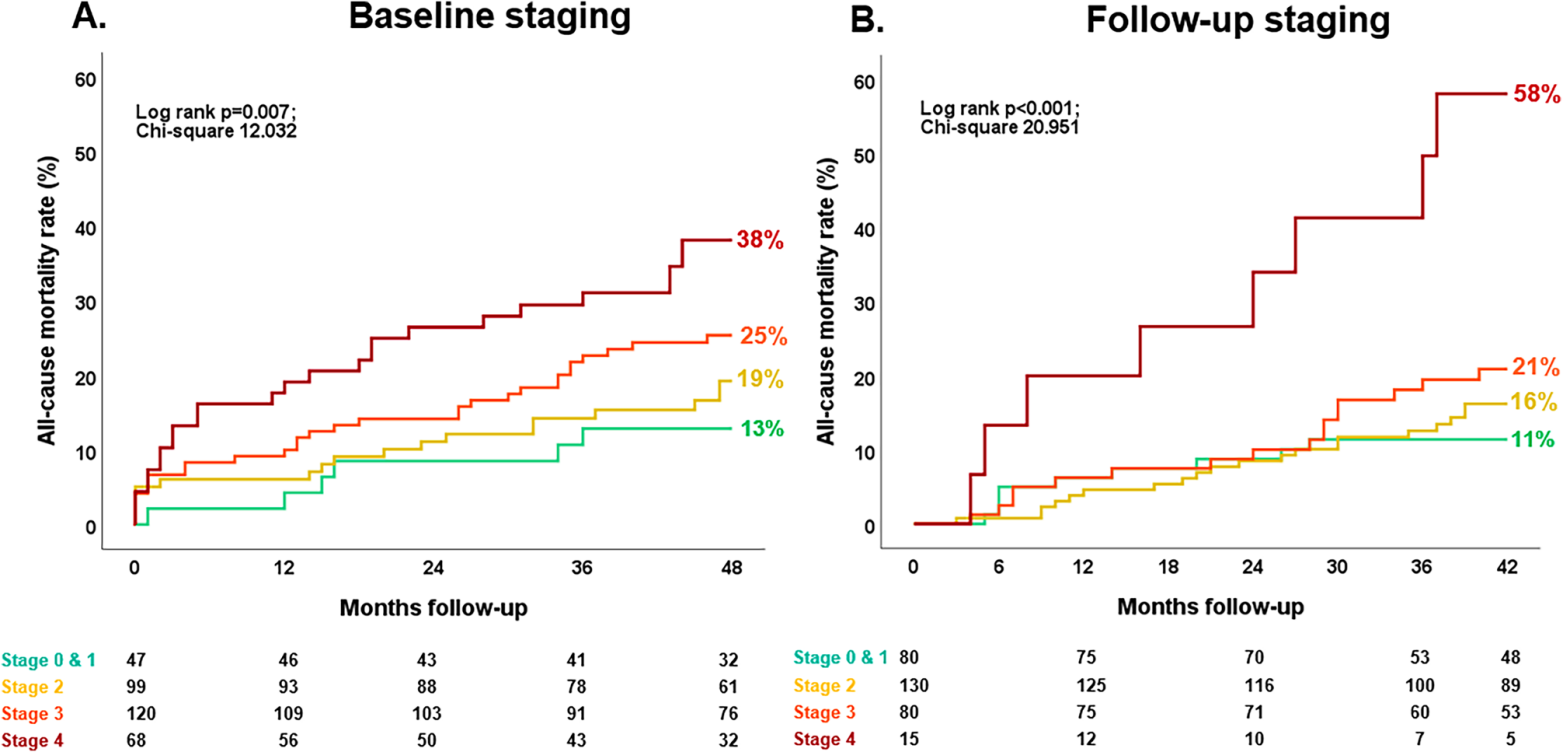
Kaplan–Meier survival curves for all-cause mortality according to cardiac damage stages at baseline (**A**) and 6-month follow-up (**B**). The figure shows the all-cause mortality rates stratified by baseline (**A**) and follow-up cardiac damage staging (**B**). A landmark analysis was performed starting from the 6-month follow-up to assess the prognostic value of cardiac damage staged 6 months after TAVI

During a median follow-up of 48 (IQR 36–48) months after TAVI, 79 patients died (24%). To assess the prognostic value of the cardiac damage staging system at baseline and 6-month follow-up, Kaplan–Meier analyses were performed according to cardiac damage stages at these two time points (Fig. 2A and B) and confirmed the ability of the staging system at baseline and follow-up to consistently stratify prognosis in women (P = 0.007 for baseline and P < 0.001 for follow-up). Notably, if RV dysfunction persisted at 6-month follow-up after TAVI, the all-cause mortality rate was substantially higher as compared to all the other cardiac damage stages (Fig. 2B). In the univariable Cox regression analysis, smoking, chronic obstructive pulmonary disease, EuroSCORE, serum hemoglobin and creatinine levels, as well as baseline and follow-up cardiac damage staging were significantly associated with mortality (Supplementary Table 3S).

In the multivariable Cox regression analysis, after adjusting for clinically significant covariates selected from the univariable analysis (Supplementary Table 3S) taking into account lack of significant collinearity (Supplementary Table 4S), each increment of baseline cardiac damage stage was associated with all-cause mortality (HR 1.537, 95% CI 1.188–1.988, P = 0.001; Model 1—Table 2). Furthermore, when adjusting for potential confounding variables, also including the baseline assessment of cardiac damage before TAVI (Model 2—Table 2), the assessment of cardiac damage at 6-month follow-up provided incremental prognostic value (HR 1.714, 95% CI 1.137–2.583, P = 0.01; Chi-square change = 6.885; P = 0.009) and this underlines the importance of reassessing the presence of cardiac damage with echocardiography during follow-up after TAVI in women to improve risk stratification.

**Table 2:** Multivariate Cox regression analysis to assess the independent associates of all-cause death

Variables	Model 1		Model 2*	
	HR (95% Cl)	P-value	HR (95% Cl)	P-value
Smoking	1.756 (1.003–3.073)	**0.049**	2.252 (1.127–4.503)	**0.022**
Chronic obstructive pulmonary disease	2.772 (1.665–4.613)	** < 0.001**	2.248 (1.208–4.182)	**0.011**
Hemoglobin, per 1 g/dL increase	0.868 (0.759–0.993)	**0.040**	0.808 (0.700–0.932)	**0.003**
Creatinine, per 1 mg/dl increase	1.079 (0.698–1.669)	0.732	1.226 (0.680–2.211)	0.497
Baseline damage staging per 1 stage increase	1.537 (1.188–1.988)	**0.001**	1.123 (0.799–1.579)	0.505
Follow-up damage staging, per 1 stage increase	–	–	1.714 (1.137–2.583)	**0.010**

Bold values represent significant P-values (< 0.05)

In baseline and follow-up staging, stages 0 and 1 combined as stage 1 due to the small number of patients in these groups

*Landmark analysis was performed when analyzing baseline and follow-up staging together

95% *CI* 95% Confidence interval, *HR* Hazard ratio, *NYHA* New York Heart Association

## Discussion

The main findings of this study, focusing on women undergoing TAVI, are as follows: first, they demonstrate that TAVI improves cardiac damage and function in women 6 months after the procedure. Second, cardiac damage staged via echocardiography, both before and 6 months after TAVI, showed consistent and independent prognostic value in women. Furthermore, echocardiographic assessment of cardiac damage at 6-month follow-up provided incremental prognostic value over baseline assessment performed before the procedure. These findings highlight the importance of using echocardiography to assess cardiac damage before and after TAVI in female patients to enhance risk stratification.

Compared with the existing literature (9, 11, 12, 22–25), the current cohort consisted only of female patients undergoing TAVI. Current guidelines recommend to consider AS-related symptoms and LV systolic dysfunction (LVEF < 50%) to set the indication for AVR in patients with severe AS (5, 26). These criteria may allow timely referral of men for AVR but could not be optimal for female patients, because women with severe AS often present with preserved LV systolic function but impaired diastolic function. In accordance with previous studies (27–29), the current findings show that women develop AS-related LV diastolic dysfunction more frequently rather than systolic function impairment, which partially improves 6 months after the procedure. In fact, it can be noted that in this cohort, consisting of only women, the prevalence of LV systolic dysfunction at baseline was relatively low (21%), whereas the majority of the cohort (≥ 70%) presented with LA dilatation and/or increased LV filling pressures (as indicated by an E/e’ ratio > 14) denoting a very high prevalence of LV diastolic dysfunction. In accordance with previous studies (6, 7), women with severe AS presented with a high prevalence of concomitant significant tricuspid regurgitation and/or increased pulmonary artery pressures (stage 3) (n = 120, 36%), and one-fifth of them had RV systolic dysfunction (stage 4) (n = 68, 20%). This is probably due to the advanced cardiac remodeling that manifested mostly as impaired LV diastolic function rather than systolic dysfunction, probably triggering late referral for TAVI, potentially limiting its beneficial effect on cardiac function (5, 26).

The comprehensive echocardiographic assessment of cardiac damage extends beyond LV systolic dysfunction, incorporating additional parameters such as LV remodeling, LV diastolic dysfunction, concomitant valvular heart disease, pulmonary hypertension, and RV function. Given the specific characteristics of AS-related remodeling in women, the staging approach could not only improve risk stratification but also help identifying the optimal timing for treatment, potentially preventing the common issue of delayed referral for valvular intervention in women. The assessment of all these echocardiographic indices rather than focusing only on LVEF may be of pivotal importance in women, where cardiac remodeling has peculiar features compared to men and LV systolic dysfunction may occur only late in the disease (29). The cardiac damage staging system, when applied before and during follow-up after TAVI, showed to be independently related with prognosis in women, suggesting the importance of follow-up not only clinically but also with echocardiography to reassess cardiac damage and improve risk stratification (30). The fact that baseline cardiac damage before TAVI was associated with long-term prognosis in this female cohort could also have important implications on the identification of the best timing for AVR in women. In fact, the cardiac damage staging system in female patients may be able to detect adverse cardiac remodeling earlier compared to the sole use of LVEF and could also trigger the indication for AVR beyond AS-related symptoms and LV systolic dysfunction (31). Acting sooner could improve outcomes of female patients and prevent the occurrence of advanced HF symptoms and irreversible cardiac damage, which could hamper the potential benefit of TAVI in women with severe AS and improve their quality of life. The hypothesis that early intervention based on echocardiographic parameters could improve outcomes is compelling but would require further validation in randomized controlled trials, potentially including a multimodality imaging approach incorporating cardiac magnetic resonance to further elucidate also the pathophysiologic mechanisms of AS-induced cardiac dysfunction (32).

## Limitations

The limitations of the present study are inherent to its retrospective single-center design. Another limitation is the exclusion of patients who died within 6 months after TAVI (9% of the initial cohort) from the paired analyses, as well as from the analysis on the prognostic value of cardiac damage assessed at 6-month follow-up. Finally, although the staging system provides a rational synthesis of cardiac damage progression in patients with severe AS, this approach could have limitations related to heterogeneous remodeling and the confounding effect of concomitant conditions (33).

## Conclusions

TAVI has a consistent beneficial effect in improving cardiac damage at 6-month follow-up in women. Cardiac damage assessed before as well as 6 months after TAVI was independently associated with prognosis, highlighting the value of assessing cardiac damage with echocardiography before and after TAVI in women to improve risk stratification.

## Supplementary Information

Below is the link to the electronic supplementary material.

Supplementary file1 (DOCX 800 KB)

## Data Availability

The data underlying this article will be shared on reasonable request to the corresponding author.

## Abbreviations

**AF**: Atrial fibrillation
**AS**: Aortic stenosis
**AVA**: Aortic valve area
**AVR**: Aortic valve replacement
**BMI**: Body mass index
**BSA**: Body surface area
**CI**: Confidence interval
**EuroSCORE**: European System for Cardiac Operative Risk Evaluation
**HR**: Hazard ratio
**LA**: Left atrium/atrial
**LV**: Left ventricle/ ventricular
**LVEF**: Left ventricular ejection fraction
**LVMI**: Left ventricular mass index
**MR**: Mitral regurgitation
**NYHA**: New York Heart Association
**PASP**: Pulmonary artery systolic pressure
**RV**: Right ventricle/ventricular
**TAPSE**: Tricuspid annular plane systolic excursion
**TAVI**: Transcatheter aortic valve implantation
**TR**: Tricuspid regurgitation
**TTE**: Transthoracic echocardiography
