# Supplementary material for: Evolution and prognostic implications of cardiac damage in women after transcatheter aortic valve implantation
Source: Int J Cardiovasc Imaging. 2025 May 22;41(7):1371–9. doi: 10.1007/s10554-025-03424-8 (PMC12241204; doi:10.1007/s10554-025-03424-8)
Supplement: Supplementary file 1 — Supplementary file1 (DOCX 800 KB) [file 10554_2025_3424_MOESM1_ESM.docx]

**Supplementary Figures.**

**Figure 1S.** **Patient flow chart.**

The current cohort study included n=334 women with severe AS who underwent TAVI for baseline analysis and n=305 women for follow-up at 6 months.

AS: Aortic stenosis; LVOT: Left ventricular outflow tract; TAVI: Transcatheter aortic valve implantation.

**Figure 2S. Proposed cardiac damage stages.**

The figure shows the echocardiography-based cardiac damage staging system, along with the specific criteria used to define each stage. The stages of cardiac damage advance from stage 0 (no damage) to stage 4 (severe damage).

LV: Left ventricular.

**Figure 3S. Evolution of cardiac damage stages.**

The figure shows the frequency of patients (n, %) who worsened (escalated at least one stage), stabilized (remained at the same stage), and improved (reversed at least one stage) at 6-month follow-up in terms of cardiac damage stages compared to baseline. Overall, the same percentages (43%) of patients improved at least one stage of cardiac damage or remained stable, underlining the beneficial effect of TAVI on cardiac remodeling.


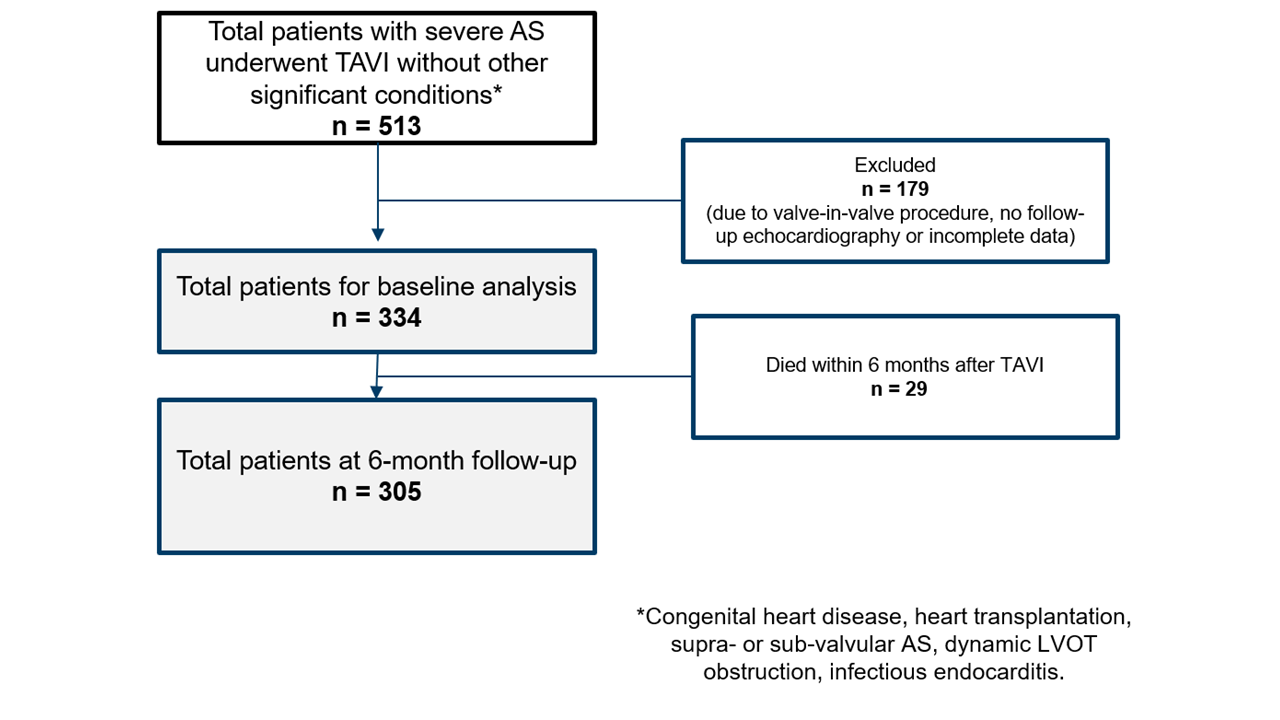


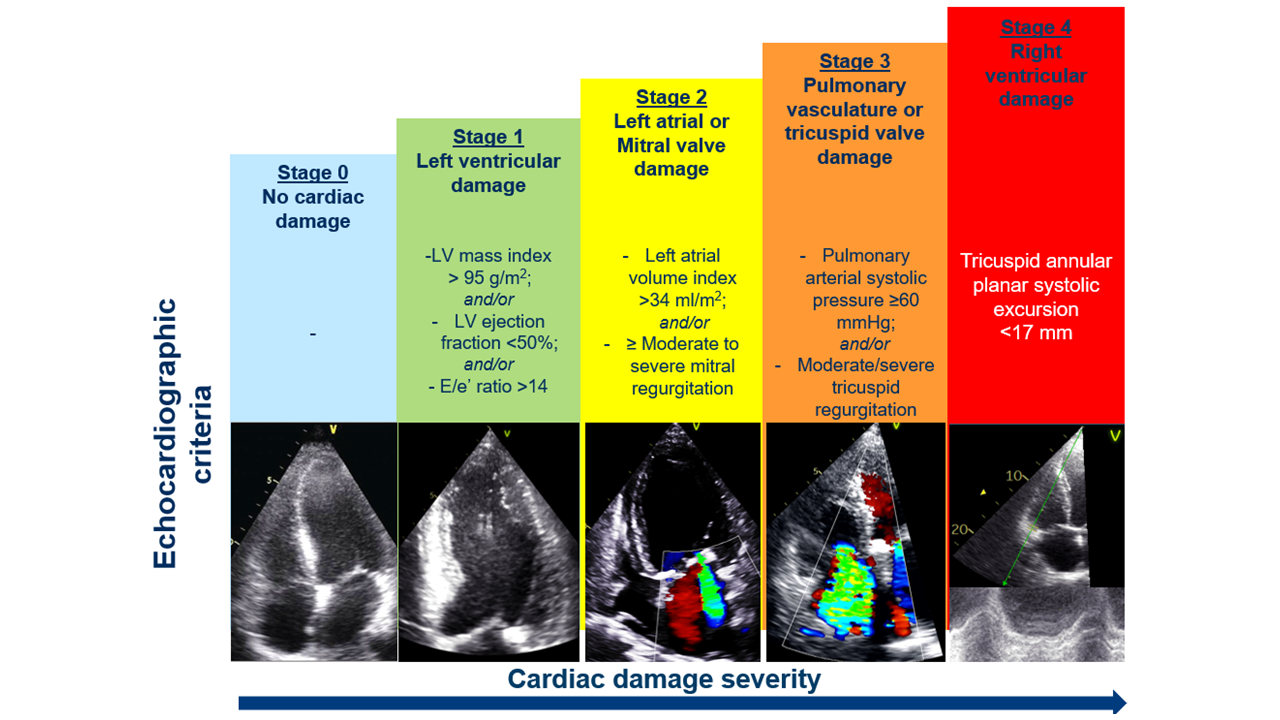

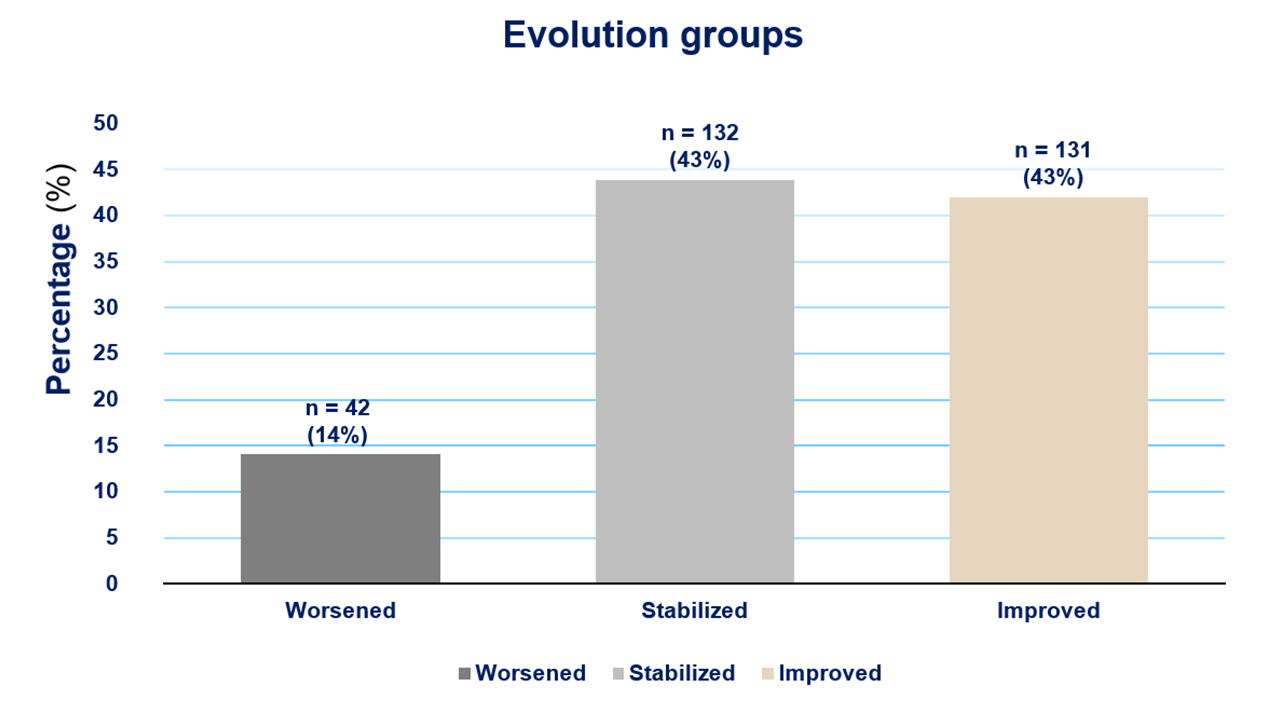


**Supplementary Tables.**

**Table 1S.** **Checklist for STROBE guideline for current cohort study.**

|  | Item No | Recommendation | Checklist |
| --- | --- | --- | --- |
| **Title and abstract** | 1 | (*a*) Indicate the study’s design with a commonly used term in the title or the abstract | 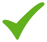 |
|  |  | (*b*) Provide in the abstract an informative and balanced summary of what was done and what was found | 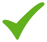 |
| Introduction | | |  |
| Background/rationale | 2 | Explain the scientific background and rationale for the investigation being reported | 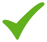 |
| Objectives | 3 | State specific objectives, including any prespecified hypotheses | 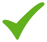 |
| Methods | | |  |
| Study design | 4 | Present key elements of study design early in the paper | 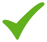 |
| Setting | 5 | Describe the setting, locations, and relevant dates, including periods of recruitment, exposure, follow-up, and data collection | 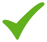 |
| Participants | 6 | (*a*) Give the eligibility criteria, and the sources and methods of selection of participants. Describe methods of follow-up | 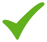 |
|  |  | (*b*) For matched studies, give matching criteria and number of exposed and unexposed | 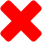 |
| Variables | 7 | Clearly define all outcomes, exposures, predictors, potential confounders, and effect modifiers. Give diagnostic criteria, if applicable | 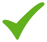 |
| Data sources/ measurement | 8* | For each variable of interest, give sources of data and details of methods of assessment (measurement). Describe comparability of assessment methods if there is more than one group | 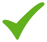 |
| Bias | 9 | Describe any efforts to address potential sources of bias | 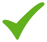 |
| Study size | 10 | Explain how the study size was arrived at | 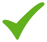 |
| Quantitative variables | 11 | Explain how quantitative variables were handled in the analyses. If applicable, describe which groupings were chosen and why | 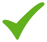 |
| Statistical methods | 12 | (*a*) Describe all statistical methods, including those used to control for confounding | 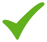 |
|  |  | (*b*) Describe any methods used to examine subgroups and interactions | 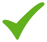 |
|  |  | (*c*) Explain how missing data were addressed | 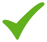 |
|  |  | (*d*) If applicable, explain how loss to follow-up was addressed | 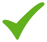 |
|  |  | (*e*) Describe any sensitivity analyses | 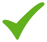 |
| Results | | |  |
| Participants | 13* | (a) Report numbers of individuals at each stage of study—eg numbers potentially eligible, examined for eligibility, confirmed eligible, included in the study, completing follow-up, and analysed | 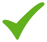 |
|  |  | (b) Give reasons for non-participation at each stage | 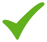 |
|  |  | (c) Consider use of a flow diagram | 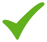 |
| Descriptive data | 14* | (a) Give characteristics of study participants (eg demographic, clinical, social) and information on exposures and potential confounders | 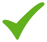 |
|  |  | (b) Indicate number of participants with missing data for each variable of interest | 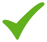 |
|  |  | (c) Summarise follow-up time (eg, average and total amount) | 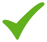 |
| Outcome data | 15* | Report numbers of outcome events or summary measures over time | 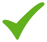 |
| Main results | 16 | (*a*) Give unadjusted estimates and, if applicable, confounder-adjusted estimates and their precision (eg, 95% confidence interval). Make clear which confounders were adjusted for and why they were included | 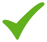 |
|  |  | (*b*) Report category boundaries when continuous variables were categorized | 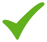 |
|  |  | (*c*) If relevant, consider translating estimates of relative risk into absolute risk for a meaningful time period | 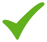 |
| Other analyses | 17 | Report other analyses done—eg analyses of subgroups and interactions, and sensitivity analyses | 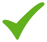 |
| Discussion | | |  |
| Key results | 18 | Summarise key results with reference to study objectives | 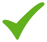 |
| Limitations | 19 | Discuss limitations of the study, taking into account sources of potential bias or imprecision. Discuss both direction and magnitude of any potential bias | 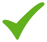 |
| Interpretation | 20 | Give a cautious overall interpretation of results considering objectives, limitations, multiplicity of analyses, results from similar studies, and other relevant evidence | 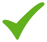 |
| Generalisability | 21 | Discuss the generalisability (external validity) of the study results | 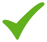 |
| Other information | | |  |
| Funding | 22 | Give the source of funding and the role of the funders for the present study and, if applicable, for the original study on which the present article is based | 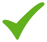 |

*Give information separately for exposed and unexposed groups.

| **Baseline clinical variables** | **Patients**  **(n=334)** |
| --- | --- |
| Age (years) | 81.3 ± 6.7 |
| **Comorbidities**  Hypertension, n (%) | 247 (76) |
| Diabetes mellitus, n (%) | 85 (26) |
| Atrial fibrillation, n (%) | 49 (15) |
| Pacemaker, n (%) | 36 (11) |
| Dyslipidemia, n (%) | 194 (60) |
| Coronary artery disease, n (%) | 153 (47) |
| Cardiac surgery, n (%) | 29 (9) |
| Myocardial infarction, n (%) | 44 (13) |
| Smoking, n (%) | 51 (17) |
| Chronic obstructive pulmonary disease, n (%) | 66 (21) |
| Peripheral artery disease, n (%) | 75 (23) |
| EuroSCORE II (%) | 3.0 (2.2-4.5) |
| **NYHA classification** |  |
| I class | 21 (7) |
| II class | 102 (32) |
| III class | 160 (51) |
| IV class | 37 (11) |
| NYHA class III or IV, n (%) | 197 (62) |
| **Laboratory parameters**  Hemoglobin (g/dL) | 12.2 ± 1.6 |
| Creatinine (mg/dl) | 0.9 (0.7-1.1) |
| **Physical parameters**  Systolic blood pressure (mmHg) | 141.7 ± 22.3 |
| Diastolic blood pressure (mmHg) | 68.0 ± 12.2 |
| Body surface area (m^2^) | 1.7 ± 0.2 |
| Body mass index (kg/m^2^) | 26.3 ± 5.0 |
| **Medication**  ACEi/ARB, n (%) | 182 (56) |
| Beta-blocker, n (%) | 189 (59) |
| Calcium antagonist, n (%) | 81 (25) |
| Diuretics, n (%) | 186 (58) |
| Aspirin, n (%) | 142 (45) |
| OAC/NOAC, n (%) | 104 (33) |
| Statin, n (%) | 182 (56) |

**Table 2S.** Baseline clinical characteristics.

Continuous variables are presented as mean ± SD or median [interquartile range]. Categorical variables are expressed as n (%).

ACEi: Angiotensin-converting enzyme inhibitor; ARB: Angiotensin II receptor blocker; EuroSCORE: European System for Cardiac Operative Risk Evaluation; NOAC: Non-vitamin K oral anticoagulant; NYHA: New York Heart Association; OAC: Oral anticoagulant.

**Table 3S.** Univariate Cox regression analysis to assess the associates of all-cause death.

| **Variables** | **Crude analysis (n=334)** | |
| --- | --- | --- |
|  | **HR (95% Cl)** | **P-value** |
| Age, per 1 year increase | 0.997 (0.964-1.032) | 0.878 |
| Body mass index, per 1kg/m^2^ increase | 0.986 (0.940-1.034) | 0.555 |
| Coronary artery disease | 1.150 (0.740-1.788) | 0.534 |
| Myocardial infarction | 1.508 (0.847-2.688) | 0.163 |
| Atrial fibrillation | 1.614 (0.932-2.794) | 0.088 |
| Cardiac surgery | 0.973 (0.448-2.115) | 0.945 |
| Diabetes mellitus | 1.135 (0.693-1.859) | 0.615 |
| Hypertension | 1.682 (0.927-3.052) | 0.087 |
| Dyslipidemia | 0.932 (0.595-1.461) | 0.759 |
| Smoking | 2.262 (1.366-3.746) | **0.002** |
| Chronic obstructive pulmonary disease | 3.345 (2.096-5.340) | **<0.001** |
| Peripheral artery disease | 1.408 (0.866-2.291) | 0.168 |
| NYHA III or IV | 1.440 (0.889-2.333) | 0.138 |
| EuroSCORE II, per 0.1% increase | 1.069 (1.023-1.116) | **0.003** |
| Hemoglobin, per 1 g/dL increase | 0.878 (0.781-0.987) | **0.029** |
| Creatinine, per 1 mg/dl increase | 1.505 (1.046-2.166) | **0.028** |
| AVA, per 0.01cm^2^ increase | 1.250 (0.621-2.516) | 0.532 |
| Baseline staging, per 1 stage increase | 1.483 (1.173-1.876) | **<0.001** |
| Follow-up staging, per 1 stage increase* | 1.587 (1.155-2.180) | **0.004** |

*Landmark analysis was performed when analyzing follow-up staging.

AVA: Aortic valve area; CI: Confidence interval; EuroSCORE: European system for cardiac operative risk evaluation; HR: Hazard ratio; NYHA: New York Heart Association.

Bold values represent significant P values (<0.05).

**Table 4S**. **Spearman’s rho cofficients**

|  | **Smoking** | **Chronic obstructive pulmonary disease** | **Hemoglobin, per 1 g/dL increase** | **Creatinine, per 1 mg/dl increase** | **Baseline damage staging per 1 stage increase** | **Follow-up damage staging, per 1 stage increase** |
| --- | --- | --- | --- | --- | --- | --- |
| **Smoking** | 1.000 | 0.211* | 0.007 | -0.014 | -0.027 | -0.077 |
| **Chronic obstructive pulmonary disease** | 0.211* | 1.000 | -0.058 | 0.096 | 0.061 | -0.017 |
| **Hemoglobin, per 1 g/dL increase** | 0.007 | -0.058 | 1.000 | -0.181* | -0.048 | 0.039 |
| **Creatinine, per 1 mg/dl increase** | -0.014 | 0.096 | -0.181* | 1.000 | 0.095 | 0.102 |
| **Baseline damage staging per 1 stage increase** | -0.027 | 0.061 | -0.048 | 0.095 | 1.000 | 0.474* |
| **Follow-up damage staging, per 1 stage increase** | -0.077 | -0.017 | 0.039 | 0.102 | 0.474* | 1.000 |

*Denotes significant correlation at the 0.01 level (2-tailed).
